# *Eucommia ulmoides Oliv*. Leaf Extract Improves Erectile Dysfunction in Streptozotocin-Induced Diabetic Rats by Protecting Endothelial Function and Ameliorating Hypothalamic-Pituitary-Gonadal Axis Function

**DOI:** 10.1155/2019/1782953

**Published:** 2019-07-29

**Authors:** Hui Fu, Xue Bai, Liang Le, Dong Tian, Hai Gao, Li-xin Qi, Ke-ping Hu

**Affiliations:** ^1^Institute of Medicinal Plant Development, Chinese Academy of Medical Sciences & Peking Union Medical College, Beijing 100193, China; ^2^Institute of Chinese Materia Medica, China Academy of Chinese Medical Sciences, Beijing 100700, China; ^3^Post-Doctoral Scientific Research Center, China Academy of Chinese Medical Sciences, Beijing 100700, China; ^4^Institutes of Biomedical Sciences, Fudan University, Shanghai 200032, China; ^5^Department of Urology, Tongji Hospital, Tongji University School of Medicine, Shanghai 200065, China

## Abstract

Erectile dysfunction (ED) is a major complication of diabetes mellitus.* Eucommia ulmoides Oliv.* is used as a traditional medicine for male impotence, but no systematic study has examined its effect on diabetes-associated ED. In this study, we investigated the effects of* Eucommia ulmoides Oliv.* leaf extract (EULE) on restoring erectile function in streptozotocin (STZ)-induced diabetic rats model. After 16 weeks of treatment, EULE administration had significantly increased intracavernosal pressure, nitric oxide (NO) levels, and cyclic guanosine monophosphate (cGMP) concentrations. Serum superoxide dismutase (SOD) and glutathione peroxidase (GSH-Px) levels were markedly higher and serum malondialdehyde (MDA) levels were lower in the EULE-treated groups than in the diabetic model group. EULE restored NO biosynthesis by significantly increasing protein kinase B (Akt) and endothelial NO synthase (eNOS) activation. Furthermore, EULE is likely to benefit the hypothalamic-pituitary-gonadal (HPG) axis, as it increased gonadotropin-releasing hormone (GnRH), follicle-stimulating hormone (FSH), luteinizing hormone (LH), and testosterone (T) concentrations as well as hormone receptors Gnrhr, Fshr, and Lhr expression levels. Hence, EULE attenuates oxidative stress, increases NO production, and activates the Akt-eNOS pathway to restore endothelial function; moreover, EULE enhances the HPG axis to improve erectile function. These results suggest that EULE may represent a new therapeutic avenue for diabetes-associated ED.

## 1. Introduction

Erectile dysfunction (ED) is defined as an inability to attain or maintain penile erection sufficient for satisfactory sexual intercourse[1]. Diabetes is a risk factor for ED; studies have shown that ED affects three out of four male patients with diabetes and this type of patient occurs earlier and three times more frequently than in the general population[2, 3].

The pathogenesis of diabetic ED is the result of multiple factors, such as advanced glycation end products (AGEs), deficiency in the nitric oxide-cyclic guanosine monophosphate (NO-cGMP) signaling pathway, oxidative stress, nervous lesions, and hypogonadism[4, 5].

Nitric oxide (NO), which is mainly derived from endothelium and nervous tissue, primarily regulates penile erection. NO stimulates the production of cyclic guanosine monophosphate (cGMP) and relaxes cavernosal smooth muscle. Declined NO synthesis and loss of NO bioactivity in the vessels are the essential features of endothelial dysfunction in diabetes[6].

Hyperglycemia has been reported to generate reactive oxygen species (ROS)[7], and data show that ROS interferes with NO production, induces cavernosal smooth muscle apoptosis, and dysregulates endothelial function[8]. In addition, accumulating evidence indicates that hypogonadism linked diabetes may play a crucial role in ED[9–11].

cGMP in the cavernosum is normally broken down by the cGMP-specific type 5 phosphodiesterase (PDE5). Selective PDE5 inhibitors are the primary treatment options for diabetic ED. However, male diabetic ED patients are more insensitive to oral PDE5 inhibitor (PDE5I) than nondiabetic patients due to neuropathy and endothelial damage[2, 12]. Therefore, new strategies to treat diabetic men with ED are needed.

In various investigations on antioxidative stress, treatments to restore NO biosynthesis and bioactivity helped to restore endothelial and erectile function in animal models of diabetic ED[8, 13, 14]. Transforming the superoxide dismutase (SOD) gene into cavernous tissue or treatment with antioxidants reduces free radical superoxide anion (O_2_^−^) production, diminishes superoxide anion levels, increases NO levels, and restores erectile response in animal models of diabetic ED[8, 15, 16]. In addition, transferring the endothelial NO synthase (eNOS) gene improved erectile function in rats with diabetic ED[17]. Therefore, reducing oxidative stress and restoring eNOS and NO bioactivity may be a potential strategy to recover erectile function in diabetic individuals.

*Eucommia ulmoides Oliv.* is used as a traditional medicine in China, Korea, and Japan[18]. Currently,* Eucommia ulmoides Oliv. *is widely used as a botanical tonic, and the water extract of* Eucommia ulmoides Oliv.* leaf, generally known as Du-zhong tea, is a popular beverage in Asia and has also been used as a new resource of medicinal and edible food against hypertension[19, 20]. Cortex Eucommia is recorded in the traditional Chinese texts Shennong Bengcao Jing and Bengcao Gangmu as being used to treat male impotence[21]. The* Eucommia ulmoides Oliv.* leaf, which contains the same components as Cortex Eucommia, has attracted attention in medical research[22].* Eucommia ulmoides Oliv.* leaf extract (EULE) exhibits antidiabetic activity in several animal models, and prior reports of its beneficial effects against hypertension and diabetes make this plant an appealing candidate treatment for diabetes-related ED. In this study, we detected the effects of EULE on erectile dysfunction in STZ-induced diabetic rats.

## 2. Materials and Methods

### 2.1. EULE Extraction and Ultra Performance Liquid Chromatography (UPLC) Fingerprint

*Eucommia ulmoides Oliv. *leaves were collected from the Beijing Medicinal Plant Garden and identified by Professor Yanze Liu from the editorial office of* Chinese Herbal Medicines*. The dried leaves were ground and extracted with distilled water for 2 h at 100°C. The supernatants were strained through a sieve, reduced pressure concentrated by a rotary evaporator below 40°C, and lyophilized. A total of 10 kg of dry leaves produced 1 kg of dry extract powder.

The chemical constituents in EULE were identified by UPLC-photodiode array (PDA) using a Waters Acquity BEH C 18 column (100mm×2.1mm, 1.7mm). The* Eucommia ulmoides Oliv.* leaf extract powder was weighed and dissolved in methanol. The composition of mobile phase is as follows: acetonitrile (solvent A) and water-trifluoroacetic acid (100:0.03, v=v) (solvent B), and the linear gradient was 0 min: 10% A; 2 min: 15% A; 5 min: 20% A; 10 min: 24% A; 15 min: 30% A; 20 min: 75% A; and 23 min: 95% A. The fingerprint of EULE was shown in Figure S1, and all the compounds were identified as per previous study [23].

### 2.2. Animals and Treatments

Sixty-five 6-week-old male Sprague-Dawley rats weighing 160-200 g were purchased from Beijing Huafukang Bioscience Co, Inc. (Beijing, China), housed in cages at the animal facility, and given access to food and water* ad libitum*. All experimental procedures were conducted conforming to Chinese animal use guidelines and were approved by the Ethics Committee of the Institute of Medicinal Plant Development (approval code: SLXD-2017022231), Chinese Academy of Medical Sciences and Peking Union Medical College (CAMS & PUMC; Beijing, China).

A control group of 10 rats was given filtered water and fed standard rat chow containing 12% fat. The other 55 rats were given filtered water and fed a high-fat diet (HFD) containing 60% fat. All rats blood glucose was normal initially, and then all rats were acclimatized for 1 month. Subsequently, the rats fasted for 14 hours. After fasting, the 55 rats received 30 mg/kg STZ (Sigma-Aldrich Chemical Co, St. Louis, MO, USA) injected into the abdominal cavity, while the control group rats received injections of equivalent volumes of citrate buffer. 72 hours after STZ or vehicle injection, tail vein blood sugar was monitored using an Accu-Chek glucose meter (Roche, Mannheim, Germany). Only STZ-treated rats with fasting blood sugar levels at or above 16.7 mmol/L were included in the diabetic group. Fifty rats met this requirement. The diabetic rats were randomly divided into five groups of 10 animals each and fed an HFD for 16 weeks. The diabetic model (DM) group was given only filtered water in addition to the diet. Sildenafil, a selective phosphodiesterase type 5 enzyme inhibitor (PDE5I), the first drug of choice for ED and was used as the positive control drug. The sildenafil group was administered 3 mg/kg sildenafil (Pfizer Pharmaceuticals Ltd, New York, NY, USA) by oral gavage[24, 25]. The other three groups included a 0.5% (w/w) EULE group, a 1% (w/w) EULE group, and a 2% (w/w) EULE group, for which EULE powder was evenly mixed into the HFD[26]. The body weights of the rats were recorded weekly.

### 2.3. Glucose Tolerance Test and Insulin Tolerance Test

A glucose tolerance test (GTT) was conducted in the 12th week after drug administration. After overnight fasting, the rats were intraperitoneally injected with glucose (2 g/kg), and blood sugar was monitored by tail prick at 0, 30, 60, 90, and 120 min using a glucose meter. An insulin tolerance test (ITT) was performed 4 days after GTT. After 4 hours of fasting, the rats were injected subcutaneously with insulin (0.75 IU/kg), and their tail vein blood sugar was measured at 0, 30, 60, 90, and 120 min.

### 2.4. *Erectile Function Measurement*

After 16 weeks of treatment, electrical stimulation of the cavernous nerve (CN) was performed to evaluate the erectile function. Rats were anesthetized by intraperitoneal injection of 10% chloral hydrate (0.35 ml/100 g) and then fixed in the supine position. Using a low midline abdominal incision, the pelvic ganglion, CN, and prostate were exposed. The skin and ischiocavernosus muscle attached to the penis was removed. To measure the intracavernous pressure (ICP), a 23-gauge scalp vein needle was connected to a PE-50 tubing filled with heparinized physiological saline (250 IU/ml) and carefully inserted into the right crus of the penis. The other side of the PE-50 tubing was linked to a Biopac Systems MP150 instrument (Goleta, CA, USA). A 23-gauge indwelling needle was carefully inserted into the left carotid artery through an incision in the side of the neck to measure systemic mean arterial pressure (MAP). The CN was identified and electrostimulated (12 Hz, 5 V, 60 s) with a stainless bipolar needle electrode. CN stimulation was conducted three times with intervals of at least 10 min between stimulations. Both ICP and MAP were monitored during electrical stimulation. Peak ICP, the ratio of peak ICP (mmHg) to MAP (mmHg), and resting ICP were recorded.

### 2.5. *Biochemical Analysis*

After the ICP experiment, the rats' blood was obtained from the abdominal aorta, then centrifuged, 3000 rpm, 10 min, 4°C. Serum was collected and stored at -80°C. Serum NO, malondialdehyde (MDA), SOD, and glutathione peroxidase (GSH-Px) levels were quantified using commercial kits (Zhongsheng Beikong Bio-technology and Science Inc, Beijing, China) according to the manufacturer's instructions. Serum levels of cGMP, gonadotropin-releasing hormone (GnRH), luteinizing hormone (LH), follicle-stimulating hormone (FSH), and testosterone (T) were determined by enzyme-linked immunosorbent kits (Sino-UK Institute of Biological Technology, Beijing, China).

### 2.6. *Histologic Examination*

After the ICP experiment, the rat penises were harvested. Three penises tissue in each group were fixed with 4% polyformaldehyde for 4 h, then embedded in optimal cutting temperature compound (OCT), cut into 5-*μ*m thick sections, and mounted on glass slides. For H&E staining, the slices were stained with hematoxylin-eosin (H&E) kit (Solarbio Science & Technology Co., Ltd., Beijing, China) according to the manufacturer's protocol. The result was shown in Figure S2.

### 2.7. *Western Blot Analysis*

Protein samples were prepared by homogenization of penile tissue in RIPA buffer (Beyotime Biotechnology, Shanghai, China) with protease inhibitor cocktail (Roche, Basel, Switzerland). After centrifugation (12000 g, 30 min, 4°C), the supernatants were collected, and then quantified by a BSA Protein Assay Kit (Huaxingbio, Beijing, China). A total of 20 *μ*g of each sample was separated by 10% sodium dodecyl sulfate polyacrylamide gel electrophoresis (SDS-PAGE) and then transferred to a poly-vinylidene-fluoride (PVDF) membrane (Bio-Rad Laboratories, Inc., Hercules, CA, USA). The membrane was blocked in TBS-T buffer (20 mmol/L Tris-HCl, 137 mmol/L NaCl, pH 7.4, and 0.2% Tween 20) containing 5% fat-free milk and shaken for 2 hours at room temperature. Then hybridized membranes overnight at 4°C with antibodies against neuronal NO synthase (nNOS), eNOS, phosphor-eNOS (Santa Cruz Biotechnology, Inc. Dallas, TX, USA), protein kinase B (Akt), phosphor-Akt, Jun N-terminal kinase (JNK), signal transducer and activator of transcription-3 (STAT3), phosphor-STAT3 (Abcam, Cambridge, MA, USA), and phosphor-JNK (Cell Signaling Technology, Inc., Beverly, MA, USA),. After washing the membrane in TBS-T three times for 15 min per wash, the membrane was hybridized to horseradish peroxidase (HRP)-conjugated secondary antibodies at room temperature for 1 hour and then rewashed in TBS-T three times. The blots were visualized with enhanced chemiluminescence (ECL). The images were scanned; the integrated density value of each protein band was analyzed with an image analysis system (Clinx Science Instruments Co Ltd, Shanghai, China).

### 2.8. *Real-Time PCR Analysis*

Total RNA was extracted from the hypothalamus, cavernosum and testes of rats using TRIzol reagent (TransGen Biotech, Beijing, China) according to the manufacturer's instructions. The purity and concentration were assayed, and cDNA was synthesized using PrimeScript™ RT Master Mix (Takara, Tokyo, Japan). Information on the primers for the target genes and the internal control gene (Actb) is listed in Table 1. The nucleotide sequences for these genes were based on NCBI reference sequences for rats and designed using Primer Premier 5.0 software. Real-time PCR was performed using SYBR® Fast qPCR Mix (Takara, Tokyo, Japan) by Applied Biosystems in strict accordance with the manufacturer's instructions. The gene expression levels are shown as the ratio of target gene expression to Actb expression. Fold changes between the groups were calculated using the 2^−∆∆Ct^ method[27].

### 2.9. Statistical Analysis

The data are shown as the mean ± SEM and were analyzed with Prism 7 software for Windows (GraphPad Software, Inc, San Diego, CA, USA). One-way analysis of variance (ANOVA) or Student's t-test was used to determine the statistical significance of the differences. If a P-value below 0.05, the difference was considered statistically significant.

## 3. Results

### 3.1. *EULE Improved Body Weight Gain, Glucose Tolerance, and Insulin Sensitivity*

**Figure 1:**
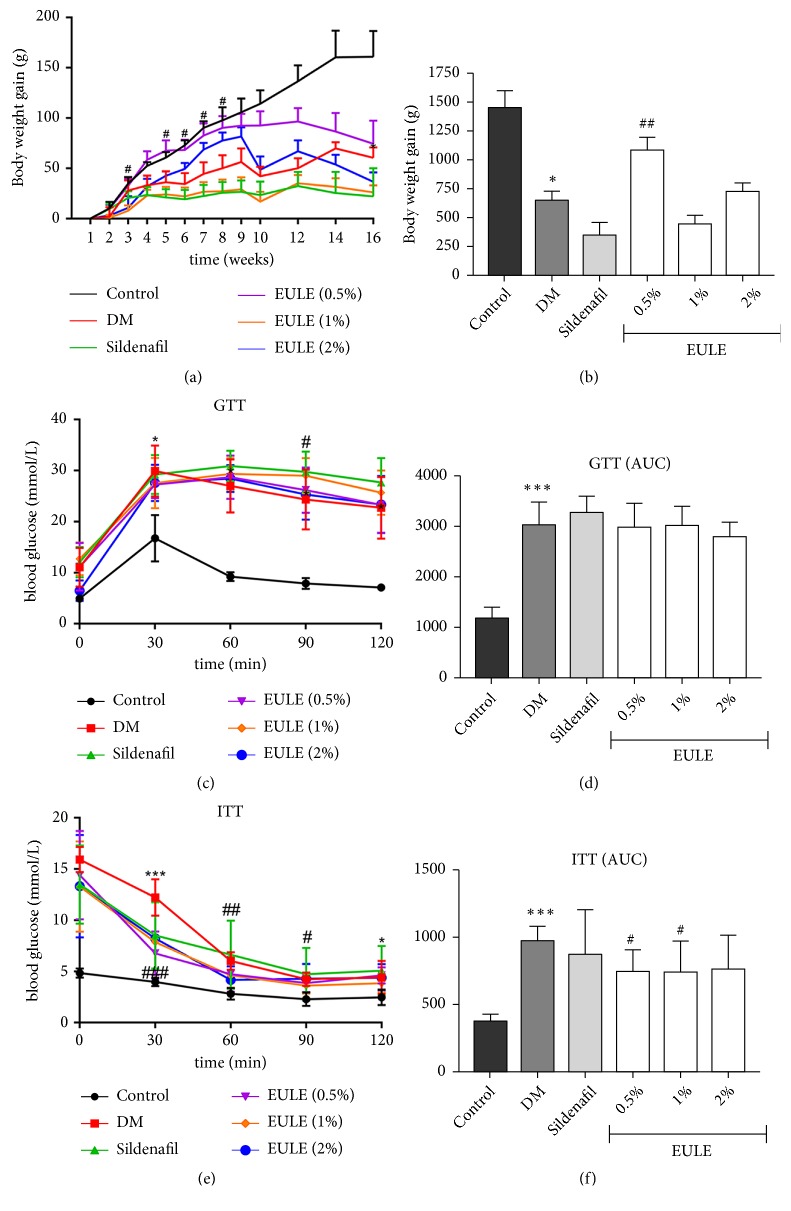
EULE improves body weight gain and glucose tolerance and prevents insulin resistance. Body weight gains (a, b) were recorded during the experiment. GTTs (c, d) and ITTs (e, f) were also performed. The data are presented as the mean ± SEM. *∗* p < 0.05 and *∗∗∗* p < 0.001 versus the control group; # p < 0.05, ## p < 0.01, and ### p < 0.001 versus the DM group (n = 10 in each group).

The body weights and blood glucose levels of the rats are shown in Table 2, and the body weight gain and the results of the GTT and ITT are shown in Figure 1. During the experimental period, body weight gain was lower in all diabetic groups than in the normal control group (Figures 1(a) and 1(b)). In addition, fasting glucose levels were significantly higher in diabetic rats than in normal control rats (Table 2). No significant diversity was found in glucose levels between DM rats and EULE-treated diabetic rats (Table 2).

The 0.5% EULE group had significantly more weight gain than the DM group during the treatment period (p < 0.01; Figures 1(a) and 1(b)), and the 0.5% and 1% EULE groups had significantly improved insulin sensitivity compared with the DM group (p < 0.05; Figures 1(e) and 1(f)).

### 3.2. *EULE Restored Erectile Function in Diabetic Rats*

**Figure 2:**
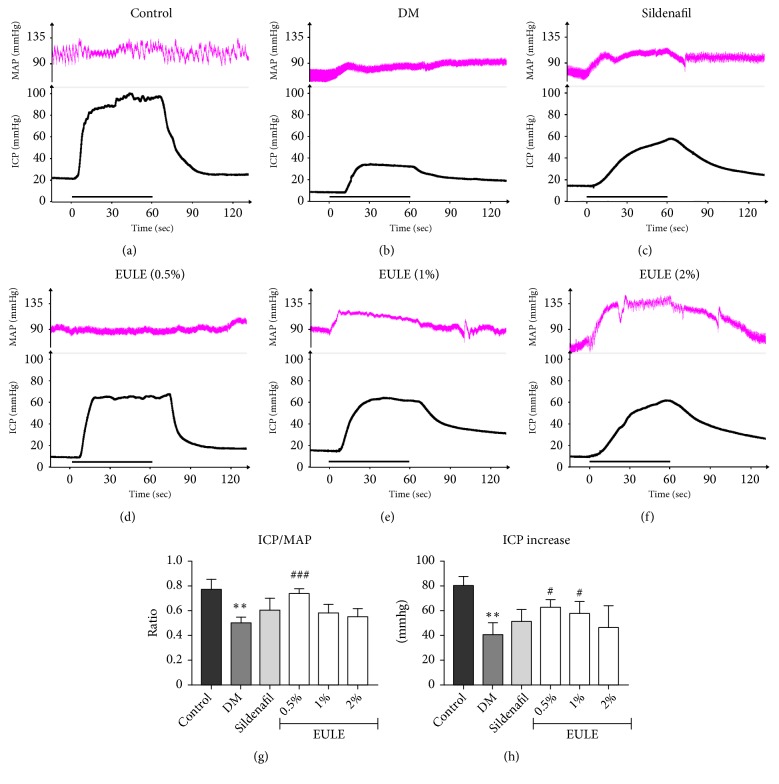
Effect of EULE on erectile function. (a-f) Curves of MAP and ICP upon electrical stimulation of the CN. The stimulus interval is indicated by a straight black line. (g-h) ICP/MAP ratios and ICP values upon electrical stimulation of the CN in each experimental group. The ICP increase was determined by subtracting the resting ICP from the peak ICP. The data are presented as the mean ± SEM. **∗∗** p < 0.01 versus the control group; # p < 0.05 and ### p < 0.001 versus the DM group (n = 5 in each group, replicated 3 times).

To determine the physiological correlation of EULE, we conducted electrical stimulation of the CN to evaluate erectile function. The changes of intracavernous pressure by stimulating the CN (5 V, 12 Hz) for 60 s in the control and diabetic rats after treatment are shown in Figures 2(a), 2(b), 2(c), 2(d), 2(e), and 2(f). The peak ICP/MAP ratios and ICP increases in rats in the DM group were significantly lower than those in the control group (p < 0.01; Figures 2(g) and 2(h)), reflecting a state of ED. Treatment with 0.5% EULE significantly improved erectile function compared with no treatment (in the DM group), as demonstrated by significant elevations in ICP/MAP ratios (p < 0.001; Figure 2(g)) and ICP increases (p < 0.05; Figure 2(h)). Treatment with 1% EULE also improved erectile function, as demonstrated by significant elevations in ICP increases (p < 0.05; Figure 2(h)).

### 3.3. *EULE Improved NO and cGMP Levels in Diabetic Rats*

**Figure 3:**
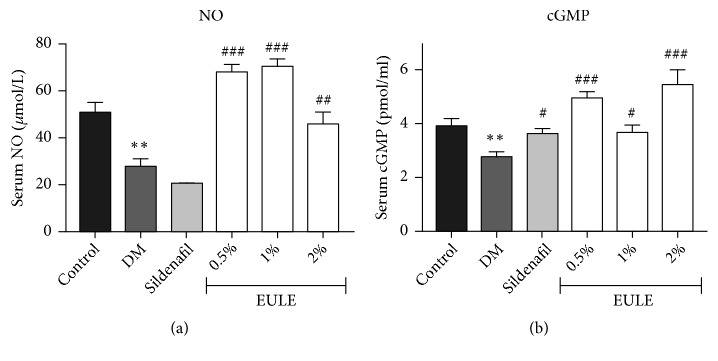
Effect of EULE on serum NO and cGMP concentrations in STZ-induced diabetic rats. The concentrations of NO and cGMP were detected with test kits. (a) NO concentrations. (b) cGMP concentrations. The data are presented as the mean ± SEM. **∗∗** p < 0.01 versus the control group; # p < 0.05, ## p < 0.01, and ### p < 0.001 versus the DM group (n = 10 in each group).

The NO and cGMP levels in rat serum were measured, and the results are shown in Figure 3. Compared with those in the DM group, serum NO and cGMP concentrations in the EULE groups were significantly increased (p < 0.05; Figures 3(a) and 3(b)). The cGMP levels in the sildenafil-treated rats were significantly higher than those in the DM rats (p < 0.05; Figure 3(b)).

### 3.4. *EULE Alleviated Oxidative Stress in Diabetic Rats*

**Figure 4:**
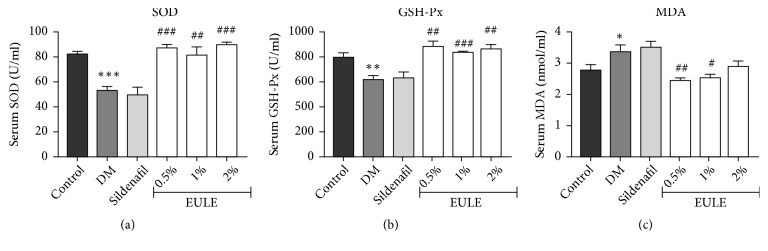
Effect of EULE on SOD, GSH-Px, and MDA concentrations in STZ-induced diabetic rats. (a) SOD concentrations. (b) GSH-Px concentrations. (c) MDA concentrations. The data are presented as the mean ± SEM. **∗** p < 0.05, **∗∗** p < 0.01, and **∗∗∗** p < 0.001 versus the control group; # p < 0.05, ## p < 0.01, and ### p < 0.001 versus the DM group (n = 10 in each group).

The levels of oxidative stress were assessed quantitatively by measuring SOD, GSH-Px, and MDA in serum using test kits. A significant increase in the MDA level and decreases in SOD and GSH-Px levels were observed in the DM group compared with the normal control group (p < 0.05, p < 0.001, and p < 0.01, respectively). The EULE-treated groups, however, showed significantly less oxidative stress than the DM group (Figure 4).

### 3.5. *EULE Restored eNOS and nNOS Protein Expression and Inhibited iNOS mRNA Level in Diabetic Rats*

**Figure 5:**
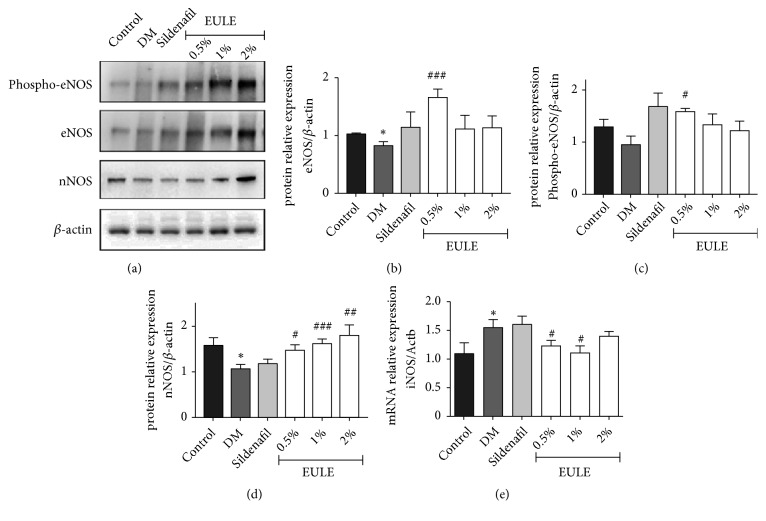
EULE restored eNOS and nNOS protein expression in diabetic rats. (a) Western blot analysis demonstrated the effect of EULE on the expression of nNOS, phosphor-eNOS, and eNOS protein in diabetic rats. (b, d) Relative expression levels of nNOS and eNOS normalized to *β*-actin expression. (c) Phosphorylated eNOS levels normalized to total eNOS levels. (e) The mRNA expression levels of iNOS was analyzed by real-time PCR and expressed as percentages of Actb levels. The data are presented as the mean ± SEM. **∗** p < 0.05 versus the control group; # p < 0.05, ## p < 0.01, and ### p < 0.001 versus the DM group (n = 10 in each group).

eNOS and nNOS protein expression in the corpora cavernosa was determined by Western blot analysis, and inducible NO synthase (iNOS) mRNA expression was determined by real-time PCR. Cavernous eNOS and nNOS expression in DM rats was significantly lower than that in normal control rats (p < 0.05; Figures 5(a), 5(b), and 5(d)). Western blot analysis showed that the EULE-treated groups had significantly greater nNOS expression than the DM group (Figure 5(d)). Moreover, 0.5% EULE significantly increased eNOS expression and induced phosphorylation of eNOS at Ser-1177 (p < 0.05; Figure 5(c)). Moreover, EULE-treated groups had less iNOS mRNA level than the DM group (p < 0.05; Figure 5(e)).

### 3.6. *EULE Increased Akt Activity in Diabetic Rats*

**Figure 6:**
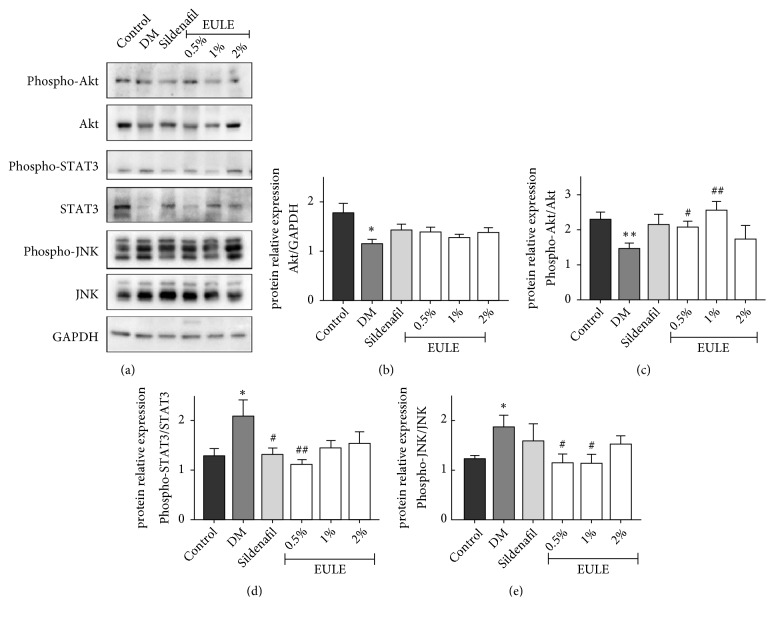
EULE increased Akt phosphorylation and suppressed JNK and STAT3 phosphorylation in diabetic rats. (a) Western blot showing the effects of EULE on the expression of Akt, phosphor-Akt, STAT3, phosphor-STAT3, JNK, and phosphor-JNK protein in diabetic rats. (b) Relative expression levels of Akt normalized to GAPDH expression. (c) Phosphorylated Akt levels normalized to total Akt levels. (d) Phosphorylated JNK levels normalized to total JNK levels. (e) Phosphorylated STAT3 levels normalized to total STAT3 levels. The data are presented as the mean ± SEM. **∗** p < 0.05 and **∗∗** p < 0.01 versus the control group; # p < 0.05 and ## p < 0.01 versus the DM group (n = 10 in each group).

The protein expression and phosphorylation levels of Akt in corpora cavernosa were measured by Western blot analysis and are shown in Figures 6(a), 6(b), and 6(c). Compared with those in the normal control group, Akt and phosphor-Akt levels were reduced in the DM group; however, treatment with 0.5% and 1% EULE significantly enhanced Akt phosphorylation at Ser-473, as shown in Figure 6(c). Moreover, the phosphorylation of JNK and STAT3 was greater in the DM group than in the normal control group, but EULE treatment attenuated this elevation in phosphorylation, as shown in Figures 6(d) and 6(e).

### 3.7. *EULE Restored Gonadotropic Hormone Secretion and Receptor mRNA Expression*

**Figure 7:**
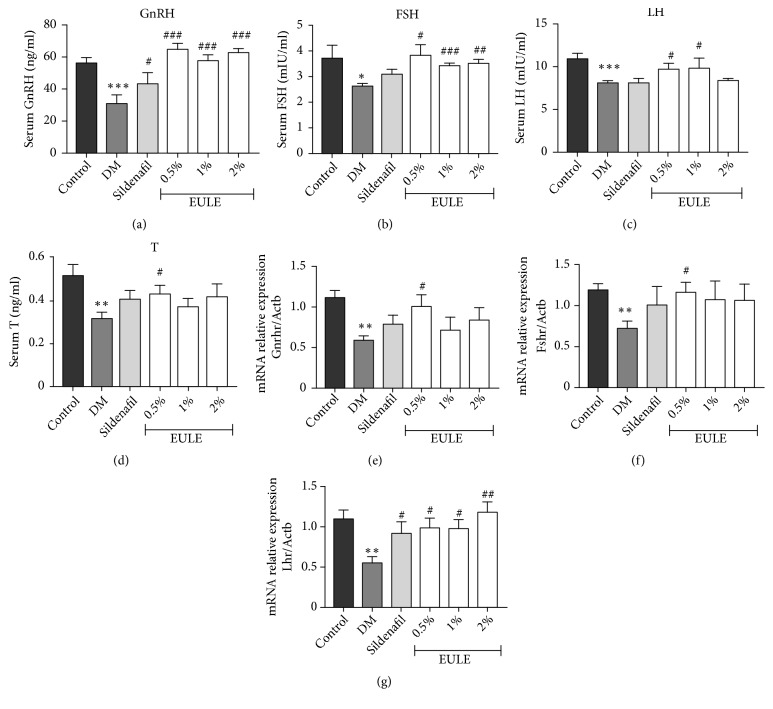
EULE restored gonadotropic hormone secretion and receptor mRNA expression. Serum levels of GnRH (a), FSH (b), LH (c), and T (d) were measured after 16 weeks of treatment. The mRNA expression levels of Gnrhr (e), Fshr (f), and Lhr (g) were analyzed by real-time PCR and are expressed as percentages of Actb levels. The data are presented as the mean ± SEM. **∗** p < 0.05, **∗∗** p < 0.01, and **∗∗∗** p < 0.001 versus the control group; # p < 0.05, ## p < 0.01, and ### p < 0.001 versus the DM group (n = 10 in each group).

The serum levels of GnRH, LH, FSH, and T were determined by enzyme-linked immunosorbent assay (Figures 7(a), 7(b), 7(c), and 7(d)). GnRH receptor (Gnrhr), FSH receptor (Fshr), and LH receptor (Lhr) mRNA levels were analyzed by real-time PCR and are expressed as percentages of normal control levels (Figures 7(e), 7(f), and 7(g)). Compared with those in the normal control group, the serum concentrations of GnRH, FSH, LH, and T in the DM group were remarkably decreased, which is consistent with observations from the clinic. Treatment with EULE significantly increased the serum concentrations of these hormones. Moreover, Gnrhr, Fshr, and Lhr mRNA expression levels were significantly greater in the EULE-treated groups than in the DM group (Figures 7(e), 7(f), and 7(g)).

## 4. Discussion

In the present study, we found that EULE treatment markedly increased the ICP/MAP ratio in diabetic rats, suggesting that EULE is able to improve erectile dysfunction in diabetic rats. At the end of the experiment, the EULE group showed significantly greater body weight gain and clearly improved tissue sensitivity to insulin compared with the DM group. This improvement may be associated with other's research that EULE exhibits antihyperglycemic activity[28]. Park et al. reported that EULE decreases glucose-6-phosphatase activity, partly alleviates hyperglycemia, and has potential therapeutic value for diabetes[29]. EULE may affect blood glucose levels through an unclear mechanism that deserves further research.

Hyperglycemia mediates increases in ROS by upregulating O_2_^−^ production and suppressing antioxidant levels in the cavernosum[30]. ROS also play a significant role in inducing apoptosis in the cavernosum. Therefore, decreasing ROS may have positive effects on diabetic ED[31]. MDA can be used as a biomarker for oxidative damage. Additionally, SOD is an enzyme that reduces O_2_^−^ and can be used to measure oxidative stress. GSH-Px is an antioxidant enzyme whose main biological role is to protect organisms from oxidative damage[32].* Eucommia ulmoides* exhibits noticeable activity as an ROS suppressant and has been found to diminish the levels of O_2_^−^ in vivo and in vitro[33–36]. Notably,* Eucommia ulmoides* leaf extract possesses stronger antioxidant activity than does raw cortex extract. Our results suggested that SOD and GSH-Px levels were significantly lower and that MDA levels were higher in the DM group than in the normal control group. These diabetes-induced changes were partly reversed in the EULE treatment group. Given the improved erectile function mediated by EULE, these results suggest that EULE improves erectile function by suppressing oxidative stress in the rat cavernosum.

Studies focusing on male diabetic ED patients have demonstrated that diabetes exacerbates the NO/cGMP signaling pathway[37]. Additionally, eNOS and nNOS-mediated cavernosal smooth muscle relaxation is impaired in diabetic animals[38]. Both clinical and basic researches have indicated that diabetes deteriorates the functional defects in the NO/cGMP pathway involved in the progression of diabetes-associated ED. In the present study, EULE-treated diabetic rats showed significantly greater NO and cGMP concentrations in the corpus cavernosum than did the DM group rats. Activation of the NO/cGMP signaling pathway is primarily, if not exclusively, required for penile erection; thus, activation of the NO/cGMP signaling pathway by EULE may be a potential mechanism by which EULE enhances erectile function.

Several reviews have reported changes in diabetes related to eNOS phosphorylation[39–41]. By increasing phosphorylation of eNOS on Ser-1177, stress promotes activation of eNOS in the cavernosum of diabetic animals. The findings of our study are consistent with those of previous studies in STZ-induced diabetic rats in which the expression levels of phosphor-Akt, an upstream mediator of eNOS phosphorylation on Ser-1177, and phosphor-eNOS (Ser-1177) were notably decreased in diabetic rats[39]. Phosphorylation of eNOS on Ser-1177 and Akt on Ser-473 coincides with the activation of these enzymes. The importance of Akt in endothelial function has been intensively investigated[42–44]. The effects of EULE in restoring cavernosal endothelial function and improving erectile function in diabetic rats seem to be mediated by phosphorylation/activation of Akt and eNOS. In addition, JNK activation is upregulated in the diabetic vasculature, and high JNK activation levels are associated with low flow-mediated vasodilation and cavernosal apoptosis, thus participating in endothelial dysfunction[45]. In our study, the EULE treatment groups showed less phosphorylation of JNK than the DM group, suggesting that inhibition of JNK activation benefits the vasculature in diabetic rats. Furthermore, it is well known that hyperglycemia and ROS activate iNOS, which damages endothelial function. STAT3 plays a crucial role in mediating iNOS expression[46]. Considerable STAT3 phosphorylation was observed in the diabetic rat cavernosum in the current study, but treatment with EULE clearly decreased STAT3 activation. Based on these results, we conclude that EULE improved diabetes-associated ED by increasing Akt and eNOS bioactivity and suppressing JNK and STAT3 activation to restore endothelial function.

Normal testosterone (T) production requires exquisite coordination of the hypothalamic-pituitary-gonadal (HPG) axis. The hypothalamus secretes GnRH in pulses, which stimulates the secretion of LH and FSH, followed by gonadal T production and spermatogenesis. Sufficient T accumulation serves as a negative feedback mechanism that decreases both GnRH and pituitary gonadotropins secretion. Acquired or congenitally deficiency in the HPG axis leads to hypogonadism[47, 48]. Studies have reported that male type 2 diabetic patients have significantly lower T levels, and hypogonadism incidence is higher in these patients than in the general population[49–51]. Study shown that T induces NO production via activation of eNOS[52]. Accordingly, T replacement therapy (TRT) improves endothelial function and prevents ED, which indicates that endogenous T can protect the cavernosal endothelium[53]. Our results showed that the diabetic rats had significantly reduced serum GnRH, LH, FSH, and T levels, consistent with the results of clinical studies[9–11]. Upon administration of EULE, the serum concentrations of GnRH, LH, FSH, and T significantly increased to levels greater than those in the DM group, strongly indicating that EULE has a beneficial effect on the HPG axis, stimulating hormone release. In addition, EULE increased the expression of receptors of the relevant hormones. This finding was unexpected and demonstrated that EULE has a stimulatory effect on the HPG axis, which may have resulted from the phytoandrogenic activity of* Eucommia ulmoides*[54]. However, the mechanisms and molecules responsible for these effects remain unclear.

## 5. Conclusions

In conclusion, the results of the present study provide evidence that EULE can improve diabetes-associated ED at both the functional and molecular levels. EULE treatment in diabetic rats can increase NO and cGMP concentrations, attenuate oxidative stress, and restore endothelial function by upregulating Akt and eNOS phosphorylation. Moreover, EULE likely stimulates the HPG axis to secrete GnRH, FSH, LH, and T and increases the mRNA expression of relevant receptors. The study indicated that EULE is a potential new therapeutic drug for diabetes-related ED.

## Figures and Tables

**Table 1:** Primer sequences used for real-time PCR.

Gene	GenBank accession	Forward primer, 5′-3′	Reverse primer, 5′-3′
iNOS	NM_012611.3	TGTTAGCCTAGTCAACTACAAGC	GTTGTTGGGCTGGGAATAG

GnRHR	NM_031038.3	CTGTCCAAAGCAAGAGCAAG	ACACATTGCGAGAAAACTGC
FSHR	NM_199237.1	CGTCATGGTATTGGGCTGGA	GCAGGCAGATGCTCACTTTCA
LHR	NM_012978.1	CGGGCTGGAGTCCATTCA	TTCTTTGGAGGGCAGTGTTTTC
Actb	NM_031144.3	ATGGTGGGTATGGGTCAGAAGG	CATGGCAGAAGAAAGACAATTA

**Table 2:** Body weights and blood glucose levels.

Index	Group	Initial	4 weeks	16weeks
Body weight (g)	Control	459.30±19.13	511.70±18.18	625.00±27.67
	DM	466.81±19.69	512.09±22.20	527.66±36.75*∗*
	Sildenafil	443.44±17.89	471.33±25.26	494.75±52.82
	EULE (0.5%)	433.55±24.06	498.77±17.57	541.66±26.17
	EULE (1%)	465.60±15.85	493.10±19.15	514.75±65.13
	EULE (2%)	478.00±16.62	511.30±16.32	576.20±17.32

Blood glucose (mmol/L)	Control	7.10±0.21	5.75±0.18	5.11±0.21
	DM	18.94±1.51*∗∗*	13.3±2.17*∗*	12.26±1.47*∗*
	Sildenafil	18.39±1.25*∗∗∗*	15.56±1.79*∗*	14.61±1.58*∗*
	EULE (0.5%)	20.66±1.60*∗∗∗*	15.36±2.63*∗∗*	14.77±1.21*∗*
	EULE (1%)	18.80±0.82*∗∗∗*	14.72±1.60*∗∗*	15.34±1.77*∗∗*
	EULE (2%)	20.59±1.69*∗∗∗*	12.44±2.15*∗∗*	12.17±1.34*∗∗*

Initial: before drug administration. 4 weeks: after drug administration for 4 weeks. 16 weeks: after drug administration for 16 weeks. Control, normal control group; DM, diabetic model group; sildenafil, diabetic group treated with sildenafil; EULE (0.5%), diabetic group treated with 0.5% EULE; EULE (1%), diabetic group treated with 1% EULE; EULE (2%), diabetic group treated with 2% EULE. Data are expressed in mean ± SEM. *∗* p < 0.05, *∗∗* p < 0.01, and *∗∗∗* p < 0.001versus control group (n = 10 in each group).

## Data Availability

The data used to support the findings of this study are available from the corresponding author upon request.

## Supplementary Materials

Supplementary Materials Figure S1: Eucommia ulmoides Oliv. leaf extract fingerprint. Figure S2: H&E staining of cavernosal tissues.
