# Supplementary material for: Eucommia ulmoides Oliv. Leaf Extract Improves Erectile Dysfunction in Streptozotocin-Induced Diabetic Rats by Protecting Endothelial Function and Ameliorating Hypothalamic-Pituitary-Gonadal Axis Function
Source: Evid Based Complement Alternat Med. 2019 Jul 29;2019:1782953. doi: 10.1155/2019/1782953 (PMC6699366; doi:10.1155/2019/1782953)
Supplement: Supplementary Materials — Figure S1: Eucommia ulmoides Oliv. leaf extract fingerprint. Figure S2: H&E staining of cavernosal tissues. [file 1782953.f1.docx]

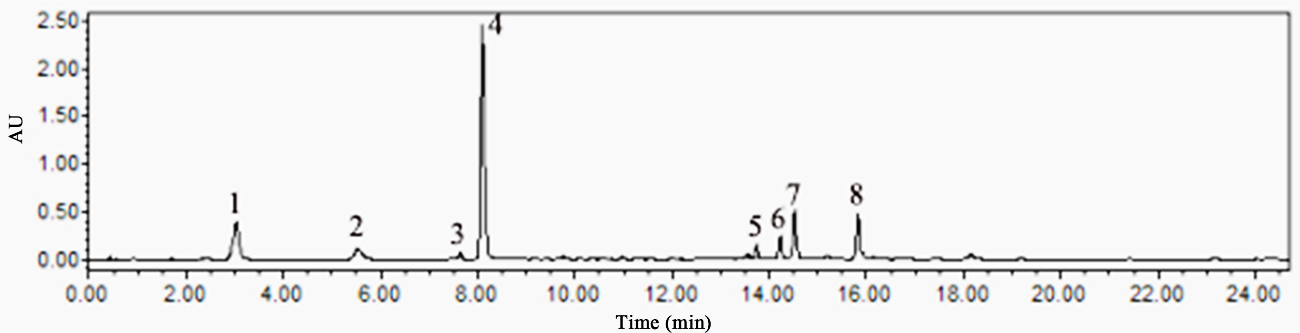


Figure S1. Eucommia ulmoides Oliv. leaf extract fingerprint. 1. Syringaresinol-di-O-glucoside;

2. Geniposidic acid; 3. Aucubin; 4. Chlorogenic acid; 5. Wogonin; 6. Quercetin; 7. Genipin;

8. Unknown


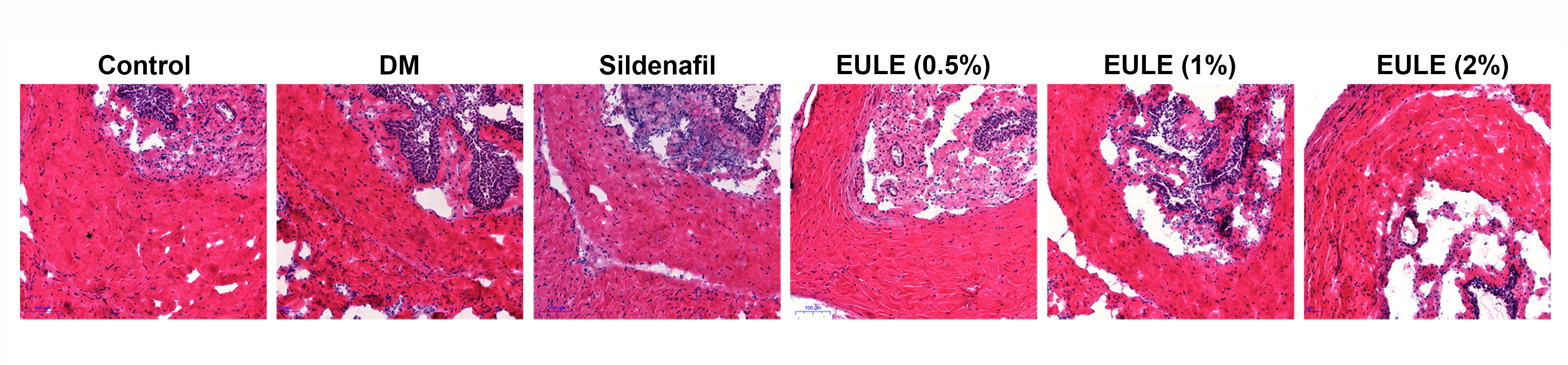


Figure S2. H&E staining of cavernosal tissues.
